# Cardiac Computed Tomography for the Assessment of Myocardial Bridging: A Scoping Review of the Emerging Role of Artificial Intelligence and Machine Learning

**DOI:** 10.3390/jcdd12090350

**Published:** 2025-09-12

**Authors:** Amro Abu Suleiman, Federico Russo, Luigi Della Valle, Davide Ausiello, Ewelina Bukowska-Olech, Vincenzo Iannibelli, M. Omar Al Droubi, Gabriella Sannino, Marco Bernardi, Luigi Spadafora

**Affiliations:** 1 Hull University Teaching Hospitals, Hull HU3 2JZ, UK; amro.a.suleiman@gmail.com; 2 Department of Clinical and Molecular Medicine, Sapienza University of Rome, 00185 Rome, Italy; luigi.della.valle.ldv@gmail.com (L.D.V.); davide.aus99@gmail.com (D.A.); 3 Division of Cardiology, Sant’ Andrea University Hospital, 00189 Rome, Italy; 4 Department of Laboratory Diagnostics, Poznan University of Medical Sciences, 60-355 Poznan, Poland; ewe.olech@gmail.com; 5 Policlinico Tor Vergata, 00133 Rome, Italy; vincenzoiannibelli@yahoo.it; 6 Walsall Healthcare NHS Trust, Walsall WS2 9PS, UK; 7 Faculty of Medicine and Surgery, Catholic University of the Sacred Heart, 00168 Rome, Italy; gabriellasannino63@gmail.com; 8 Department of Medical-Surgical Sciences and Biotechnologies, Sapienza University of Rome, 00185 Latina, Italy; marco.bernardi23@gmail.com (M.B.); luigispadafora167@gmail.com (L.S.); 9 Division of Cardiology and Interventional Cardiology, Santa Maria Goretti Hospital, 04100 Latina, Italy

**Keywords:** myocardial bridging, artificial intelligence, machine learning, cardiac computed tomography

## Abstract

(1) Background: Myocardial bridging (MB) is a congenital coronary anomaly with potential clinical significance. Artificial intelligence (AI) applied to cardiac computed tomography angiography (CCTA), particularly through CT-derived fractional flow reserve (CT-FFR), offers a novel, non-invasive approach for assessing MB. (2) Methods: We conducted a systematic review of the literature focusing on studies investigating AI-enhanced CCTA in the evaluation of MB. (3) Results: Ten studies were included. AI-based models, including radiomics, demonstrated moderate to high accuracy in predicting proximal plaque formation, and motion correction algorithms improved image quality and diagnostic confidence. Other findings were limited by the types of studies included and conflicting findings across studies. (4) Conclusions: AI-enhanced CCTA shows promise for the non-invasive functional assessment of MB and its risk stratification. Further prospective studies and validation are required to establish standardized protocols and confirm clinical utility.

## 1. Introduction

Myocardial bridging (MB) is defined as an intramyocardial segment of an epicardial coronary artery [1]. Although MB can theoretically involve any segment of the coronary tree, the majority (approximately 60%) occur in the left anterior descending artery (LAD) [2]. Myocardial bridges are typically classified as superficial or deep, depending on the anatomical arrangement and depth of myocardial fibers. The true prevalence of MB remains a subject of debate, as it varies significantly based on the definition applied and the modality used for identification, including autopsy, computed tomography (CT), or intravascular ultrasound (IVUS) [1]. Notably, the frequency of MB in the LAD is reported to exceed 50% in autopsy studies, whereas it is identified in fewer than 5% of cases by angiography [3,4].

The advent of multi-detector CT has markedly improved the detection rate of MB, particularly when the myocardial bridge is greater than 1 mm in thickness, yielding a sensitivity comparable to that of autopsy studies [5]. Although the majority of cases are asymptomatic, MB has been associated with angina, exertional dyspnea, ventricular arrhythmias, syncope, and, in rare cases, sudden cardiac death [6,7]. In the majority of cases, it is an incidental finding with 97% survival at 5 years. These pathologic associations are typically seen in patients with associated myocardial ischemia and/or concurrent atherosclerosis [8].

Invasive coronary angiography (ICA) remains an important diagnostic tool, although its sensitivity for detecting MB is relatively low, at approximately 5% [3]. The use of IVUS has enhanced the sensitivity of ICA in detecting systolic compression within a bridged segment [9]. ICA may also be used for functional assessment of MB using fractional flow reserve (FFR). Invasive FFR (iFFR) has emerged as a gold standard technique for assessing coronary lesions following evidence in the FAME [10] and DEFER [11] trials showing improved outcomes in the setting of PCI. Despite this, the evidence is not as strong in the context of myocardial bridging, in which there is dynamic rather than fixed obstruction of coronary blood flow. Dobutamine-stress diastolic FFR (dFFR) has been considered as the reference standard for the physiologic assessment of MB, given its increased ability to identify hemodynamically significant MB when compared with standard FFR [12]. However, these techniques are more invasive than standard coronary angiography and are not feasible for use in the routine evaluation of patients.

Management of MB is typically conservative, with a focus on treating anginal symptoms and associated coronary artery disease (CAD). Medical treatments include beta-blockers due to their ability to reduce heart rate and contractility while prolonging diastolic coronary filling. Dihydropyridine calcium channel blockers may be considered as adjunct therapy, given their vasodilatory effects [13]. In cases where symptoms are refractory to medical management, surgical intervention, such as myotomy or coronary artery bypass grafting (CABG), may be warranted. Myotomy is generally preferred in the absence of concomitant obstructive coronary artery disease (CAD) due to the risks associated with CABG, including graft failure secondary to competitive flow [14]. Stent implantation is not routinely performed because of high rates of in-stent restenosis and stent fracture [15,16].

## 2. CCTA and Myocardial Bridging

CCTA, equipped with its multiplane and three-dimensional functionalities, has notably increased the detection of MB. Inevitably, many of these will be incidental findings. However, the clinical relevance of MB is often unclear, and functional assessment remains key in the accurate detection of hemodynamically significant bridging, which is necessary to guide adequate treatment. Functional and dynamic studies are, therefore, of utmost relevance.

Current strategies used for the assessment of myocardial ischemia include invasive FFR, cardiac magnetic resonance (CMR), myocardial perfusion imaging (MPI), nuclear MPI, and dynamic CT-MPI. The latter has been established in the literature as an accurate method for diagnosing myocardial ischemia when compared with the former techniques [17,18,19,20], highlighting the utility of CT in cardiac imaging, and it represents an alternative to invasive assessment, nuclear MPI, and CMR-MPI. These tools are well evidenced in the setting of atherosclerotic disease, with a less established role in the assessment of MB-related ischemia.

Standard CCTA, being primarily an anatomical assessment, has limitations in evaluating the functional consequences of MB. While CCTA can depict systolic narrowing of the lumen, it does not directly quantify the hemodynamic impact of the MB on myocardial perfusion.

The addition of alternative techniques can enhance CCTA. For example, stress–rest dynamic CT-MPI can be used to assess the hemodynamic significance of MB. CCTA, in conjunction with stress–rest dynamic CT MPI, can allow for the detection of MB and the evaluation of functional significance, and it can ultimately guide patient management in a “one-stop shop” examination, as described by Schicchi et al. [21] The advantage of this is that, once MB is identified on CT, the same modality can then be used to assess the bridge without the patient needing to undergo secondary imaging via SPECT/CMR or ICA. The total patient time is about 30 min. Moreover, CT-MPI offers a lower mean dose of radiation (6.8 mSv) as compared to SPECT (10.4 mSv). Given that MB is a commonly encountered finding on CCTA, the ability to promptly assess the relevance of such findings has the potential to reduce costs and harm to patients and allows earlier effective treatment. These techniques, however, have never been validated in the context of MB-related ischemia, and their use remains non-guideline backed and not entirely evidence-based due to a lack of rigorous prospective studies and controlled trials.

Novel techniques are emerging, including the use of artificial intelligence (AI) and machine learning (ML) in conjunction with CCTA, that may improve the assessment of MB. Given the challenges associated with an ever-increasing volume of imaging studies and the associated incidental diagnoses of MB, innovative tools will be required to accurately assess the significance and implications of these lesions and guide both the need for treatment and appropriate treatment strategies. The increased clinical complexity poses a challenge to radiologists and clinicians in what to do with these lesions; on the part of the radiologist—whether to report on them or not—and on the part of the clinician—what to do with this information once reported on. It would be unfeasible, both in terms of costs to healthcare systems and due to time and utilization of facilities, and indeed harmful to offer invasive assessment to this increasingly large number of patients with MB identified on imaging. The above-described “one-stop shop” examination poses an ideal solution to this problem, though one that remains not yet feasible in clinical practice.

## 3. Artificial Intelligence and Machine Learning in CCTA

Artificial intelligence (AI) and machine learning (ML) are emerging technologies that are becoming pivotal in advancing cardiac imaging, particularly in the context of CCTA. These technologies enhance image acquisition, segmentation, anatomical detection, motion correction, quantitative analysis, and evaluation of functional significance, addressing the increasing complexity and volume of cardiovascular imaging data [22,23].

Even though the terms AI, ML, and deep learning (DL) are often used synonymously, they are fundamentally hierarchical [24]. Artificial intelligence can be broadly divided into machine learning and intelligent computing. ML is the main technology of AI, which includes supervised learning, unsupervised learning, and deep learning.

Supervised learning involves training algorithms with labeled data, allowing the model to learn from known examples and predict outcomes for new data. Common supervised learning methods include the following:

- Artificial neural networks (ANNs) are computational models inspired by biological neurons that can learn complex patterns; in cardiac CT, they may be used to identify subtle morphological signs of myocardial bridging.
- Support vector machines (SVMs) are algorithms that find the optimal boundary separating different classes of data; they are useful in distinguishing stenosed/bridged coronary segments based on extracted image features.
- Decision trees—flowchart-like models that split data based on decision rules; they can be applied to classify stenosis/bridging severity based on vessel compression metrics.
- Random forest (RF) is a combination of decision trees that improves accuracy and model stability by aggregating predictions of multiple decision trees at the cost of higher processing time—it can be used to integrate multiple CT-derived parameters for diagnosis.
- A Naive Bayes classifier is a probabilistic method based on Bayes’ theorem, assuming independence between features, which may help in rapid classification.
- K-nearest neighbor (k-NN) is an algorithm that classifies data points based on the majority label of their closest neighbors/similarity to known examples; it is useful for matching new CT images with a library of annotated MB cases.

Unsupervised learning uses unlabeled data to identify hidden patterns, mainly through clustering algorithms (grouping similar data points) and association rule algorithms (finding relationships between variables). In cardiac CT, this can be performed through grouping cases with similar coronary motion profiles and identifying feature combinations linked with MB.

DL is a subset of ML that uses multi-layered neural networks to automatically learn hierarchical features from raw data. Common DL architectures include the following:

- Convolutional neural networks (CNNs) are used for processing grid-like data, including images, making them relevant to cardiac CT analysis. CNNs can segment coronary arteries and detect vessel compression.
- Recurrent neural networks (RNNs) are designed to handle sequential or time-series data; they are potentially useful for analyzing dynamic CT datasets or cinematic reconstructions.
- Deep neural networks (DNNs) are multi-layered models that can learn highly complex and non-linear relationships, for example, integrating CT imaging data with clinical parameters for a more comprehensive assessment of MB.

AI technology differs in its applications and limitations for different data types. Therefore, the accurate diagnosis of coronary artery disease can only be achieved by finding an appropriate intelligent mathematical model to match the CCTA imaging data [25].

The role of AI in the evaluation of MB is still experimental and under study, but there are established benefits of AI in CCTA. AI algorithms, especially CNNs, can process large datasets rapidly, constructing images faster than traditional methods. CNNs enable automatic segmentation of coronary arteries, identification of systolic compression, and estimation of functional indices, with high accuracy and direct image-based learning compared to traditional supervised models, such as random forests or support vector machines, which require manual feature extraction. CNNs offer superior performance in tasks relevant to MB by leveraging large imaging datasets and learning directly from pixel-level data [22,26,27].

In the context of myocardial bridging (MB), AI-enhanced coronary CT angiography (CCTA) offers unique opportunities to improve both image quality and diagnostic confidence. Adaptive algorithms can tailor acquisition parameters to the individual patient, producing clear, high-resolution views of the bridged segment while limiting radiation exposure. Automated segmentation of coronary arteries helps isolate and characterize the intramyocardial course, while integrated calcium scoring and risk stratification provide additional context on overall coronary health. AI can also facilitate multi-planar and dynamic reconstructions, enabling detailed visualization of systolic compression and its spatial relationship to surrounding myocardial tissue [28].

## 4. Artificial Intelligence and Machine Learning in Myocardial Bridging

**Figure 1:**
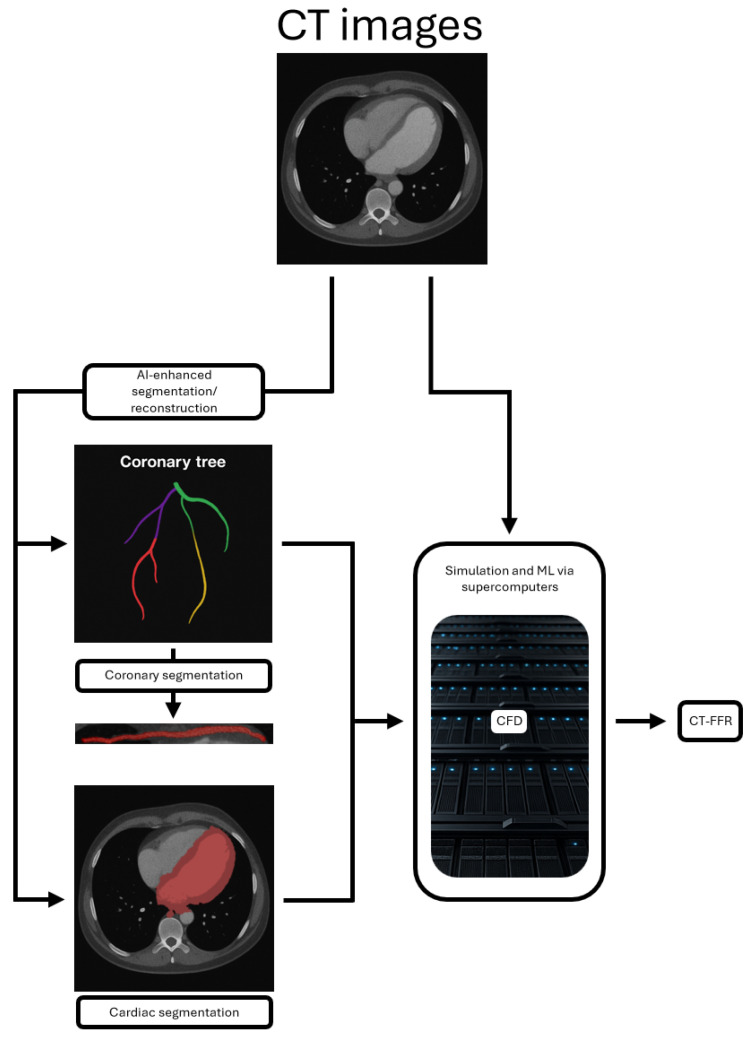
Illustrative workflow demonstrating the utility of AI-enhanced segmentation and reconstruction, CFD, and CT-FFR.

Specific techniques of interest in assessing MB are depicted in Figure 1 and listed in Table 1. Namely, these are:

- AI-enhanced segmentation and reconstruction.
- Computational fluid dynamics (CFD), which predicts blood flow dynamics and hemodynamic effects.
- CT-derived fractional flow reserve (CT-FFR), a ML technique which is particularly exciting and has already been established as a tool for assessing the functional significance of stenotic coronary lesions as an alternative to invasive FFR.

### 4.1. AI-Enhanced Segmentation and Reconstruction

Heart segmentation is the process of delineating different anatomical structures of the heart from medical imaging techniques such as MRI, CT scans, and echocardiography [29]. The method relies on fully connected neural networks (FCNNs), such as U-Net [30]. AI-enhanced segmentation enables detailed analysis of the heart’s four chambers, valves, arteries, and veins, as well as the identification of pathophysiological lesions such as myocardial infarction, ischemia, and cardiomyopathy. This facilitates pre-surgical planning, device design, and real-time monitoring of heart disease [31]. Traditionally, heart segmentation was performed manually, which was both time-consuming and labor-intensive. The introduction of automated analysis has reduced error rates and hastened clinical diagnosis. As a result, AI-enhanced segmentation is now widely adopted in cardiac clinics, aiding in the evaluation of cardiac function, wall thickness, and other key risk assessment metrics [29]. Reconstruction algorithms and techniques can be used to enhance image quality, perform motion correction, identify structures of interest [32,33], and streamline workflows by automating post-processing and analysis, including functions such as cross-sectioning, volume rendering, multi-planar reformation, and curved planar reformation techniques [34]. Specific uses of these functions and applications to MB have been demonstrated in several studies [35,36,37,38], including motion correction models for improving image quality, automated reconstruction and segmentation of coronary arteries, coronary stenosis/plaques, and identification of bridged segments.

### 4.2. Computational Fluid Dynamics (CFD)

CFD enables precise modeling of intracoronary hemodynamics and has numerous applications in cardiology. For instance, it is used to create patient-specific 3D coronary artery geometries, allowing visualization of specific vascular segments with a resolution of approximately 1 mm or lower. These reconstructions are derived from imaging data such as invasive coronary angiography (ICA), computed tomography coronary angiography (CTCA), intravascular ultrasound (IVUS), and optical coherence tomography (OCT). The selected vascular segment is then subdivided into smaller elements (meshing), and a mathematical model is applied to solve the fluid motion equation. These simulations help evaluate blood velocity and pressure gradients within arteries, wall shear stress, which is a factor in plaque formation and progression, and microvascular resistance, which allows ischemic assessment [39,40,41,42]. CFD is also used in transcatheter aortic valve implantation (TAVI) for pre-procedural planning, device optimization, and post-procedural assessment, including hemodynamic evaluation, valve size, and positioning, paravalvular leak prediction, left ventricular workload, and coronary perfusion impact [43]. With regard to MB, CFD has been used experimentally to study and model the hemodynamic impact of MB by combining ICA and IVUS data [44,45]. A more practical and utilized application of CFD is in CT-FFR.

### 4.3. CT-Derived Fractional Flow Reserve (CT-FFR)

CT-FFR is a CFD-based method using ML and computational modeling in conjunction with data from CCTA to estimate FFR [46]. Current evidence suggests that CT-FFR is a promising alternative to invasive FFR, the current gold standard for evaluating coronary lesion-specific ischemia [47,48,49]. Trials (DISCOVER-FLOW, NXT) have demonstrated the diagnostic accuracy and discrimination of CT-FFR when compared to invasive FFR for the assessment of CAD [50,51]. The most used and the only U.S. Food and Drug Administration (FDA)-approved method is HeartFlow, which applies CFD with remote supercomputers. It creates a personalized three-dimensional (3D) model of the coronary arteries using semiautomatic contouring and segmentation. Next, the model is refined based on the patient’s blood flow conditions, including viscosity and pressure conditions. Myocardial blood flow is proportional to myocardial mass, whereas microvascular resistance is inversely related to the size of the epicardial coronary arteries. The model eliminates the need for adenosine infusion as it also accounts for the reduction in microvascular resistance caused by adenosine [52,53]. The use of CT-FFR in the assessment of MB is less robust and guideline-based than its use for CAD. Evidence regarding its use for the functional assessment of MB-related ischemia remains lacking, though data on its use in this context are emerging in the last few years and are discussed below. CT-FFR computation can be performed in the systolic and diastolic phases. In addition, measurements may be taken at different points—most often proximal and distal to the MB/stenosis, from which ΔCT-FFR is derived as the difference between CT-FFR measurements taken at two points.

## 5. Challenges in Practical Implementation

While AI and machine learning (ML) offer considerable advantages for cardiac CT, their integration into routine myocardial bridging (MB) assessment is not without obstacles. Variability in imaging protocols, diagnostic thresholds, and reporting standards between institutions can make it difficult to apply AI tools consistently. Without harmonized reference criteria for AI-assisted CCTA interpretation, achieving reproducible results across centers remains challenging.

Robust AI performance depends on high-quality, diverse datasets, yet many algorithms are trained on limited or homogeneous image collections. This raises concerns about generalisability to different patient populations, scanner technologies, and clinical contexts. For MB, where subtle anatomical and functional changes may influence diagnosis, narrow training datasets risk underrepresenting relevant variations. Collaborative data-sharing initiatives and standardized imaging protocols are, therefore, essential to develop algorithms capable of performing reliably across diverse settings [54].

Technical issues such as overfitting, class imbalance, and limited model explainability also hinder clinical adoption, introducing the potential for bias. Human oversight is indispensable; radiologists must review and validate AI-derived outputs to ensure accuracy and maintain patient safety. Ethical and regulatory considerations, particularly those concerning patient privacy, become even more pressing in data-driven healthcare systems. ML and deep learning models often require extensive personal health information to generate predictions, making strict adherence to privacy safeguards and compliance frameworks mandatory. Progress in this field will depend on sustained collaboration between clinicians, AI developers, and regulatory bodies to ensure that technological innovation enhances, rather than replaces, clinical expertise [28,29].

Currently, there is a lack of data on the real-world application of these technologies and techniques with regard to the assessment of MB. We, therefore, conducted a systematic review of the literature, aiming to highlight currently available evidence and identify gaps in the literature and areas for future research.

## 6. Methods

A comprehensive electronic search of the literature was conducted from inception to 17 April 2025 in the following databases: Embase, Medline, PubMed, and Cochrane. A keyword search was performed using the terms “myocardial bridging”, “myocardial bridge”, “tomography”, “computerized tomography”, “computerized tomography angiography”, and Boolean operators (AND, OR), resulting in a total of 1159 studies.

Semi-automated screening of the literature search results was performed by two authors using “Covidence”, an online systematic review tool, in a two-phase process. The first phase consisted of title/abstract screening for potentially eligible studies. The second phase consisted of the retrieval and assessment of full texts for eligible studies.

Studies were included if (1) the population being studied was patients with myocardial bridging, (2) the study incorporated the use of any AI/ML techniques in its methodology, and (3) they were English-language studies published in peer-reviewed journals.

Studies were excluded if (1) they were published in a language other than English, (2) the population studied was not patients with myocardial bridging, or (3) the study did not employ the use of AI/ML techniques.

**Figure 2:**
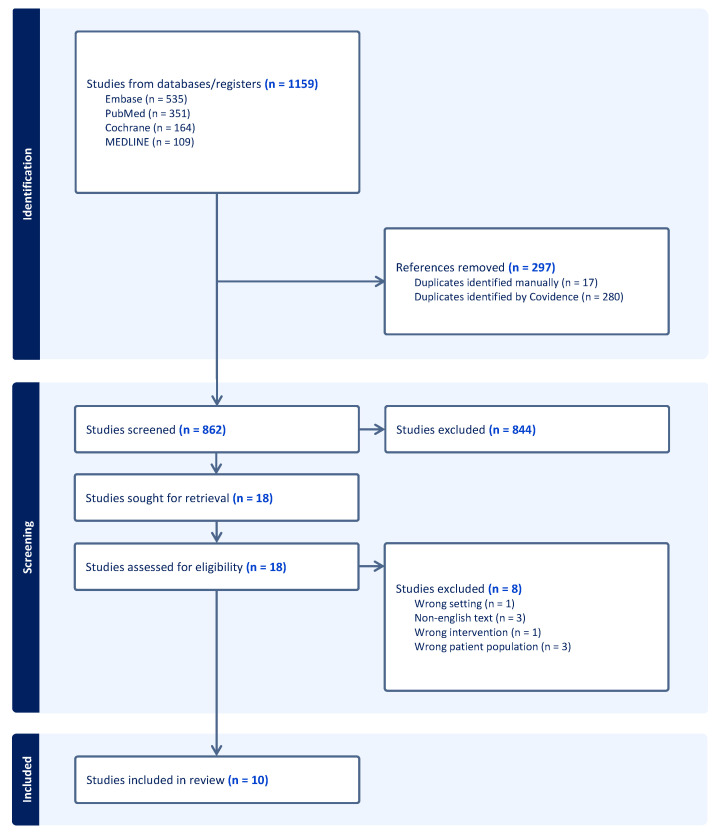
PRISMA flow diagram detailing the study selection process.

Data from eligible studies were extracted using a data extraction template containing information on the study characteristics, participants, imaging modality and protocol, AI/ML technique used, and results. Automatically screened duplicates were then manually verified by the reviewers. A full diagram detailing the study selection process is shown in Figure 2.

Given the high degree of heterogeneity between studies in terms of design, aim, methodology, study population, and the fact that none of the included studies are randomized control trials (with the majority being retrospective studies with the exception of one case report), our review reports on the findings of individual studies rather than analysis of results due to non-comparability between studies. We summarize the available literature on the topic to date and highlight areas for further research.

## 7. Results

A total of 10 studies were included in the review. These are summarized below (Table 2).

## 8. Discussion

This systematic review synthesized evidence from 10 studies evaluating the application of AI in CCTA for the assessment of MB. Collectively, these highlight that AI-based techniques are logistically feasible in practical applications for this purpose. In particular, automation and reconstructive techniques were shown to be useful across several studies, with authors reporting improvements in image quality as well as utility in optimizing certain aspects of workflow [35,36,37,60]. In addition, AI-driven techniques, particularly CT-FFR, can provide functional insight beyond static anatomical measurements. For the purpose of discussion, CT-FFR-derived measurements will be summarized as a group (including systolic/diastolic CT-FFR and ΔCT-FFR measurements).

Positive findings included significant association between CT-FFR values and MB with atherosclerosis compared to MB alone [36]; significant association between LAD stenosis severity and CT-FFR values [57,59]; a role in prediction of plaque formation in MB [56,59]; correlation with iFFR in one study [38]; correlation with symptoms of chest pain and angina in a second study [56]; correlation between CT-FFR and certain anatomical features of MB (depth, length, distance from aorta) [35,56]; high negative predictive value (NPV) of CT-FFR [35,59] and high positive predictive value (PPV) in MB with >70% proximal stenosis [38]; and improved image quality [37].

Eight studies utilized CT-FFR, with studies focusing on the following various aspects of its use: (1) assessment of functional ischemia in MB; (2) prediction of proximal atherosclerotic plaque formation; (3) association with certain anatomical characteristics of MB; (4) correlation with symptoms; (5) correlation with a reference measurement (CT-MPI, invasive assessment methods); and (6) comparison of different CT-FFR derived measurements.

However, there were conflicting findings across several studies (despite variations in design, inclusion/exclusion criteria, and methods), and several of the above-mentioned findings were not verified in other studies.

For example, Zhou et al. [38] reported high concordance between CT-FFR and iFFR in patients with LAD MB and atherosclerosis, while Jubran et al. [58] reported that CT-FFR values did not correlate with iFFR values in their study. It is important to note that in these two retrospective studies, the study design and study population differed. Jubran et al. [58] selected patients with ≤50% coronary artery stenosis on ICA, while Zhou et al. [38] selected patients who had undergone CCTA and subsequently underwent ICA, irrespective of the degree of coronary artery stenosis identified. Moreover, Jubran et al. [58] employed a less rigorous study design without identification of a clear cohort, and the number of patients who underwent CT-FFR and those who underwent iFFR was not 1:1 (49 patients underwent CT-FFR, with only 28 patients undergoing iFFR).

Two studies attempted to correlate CT-FFR data with clinical data in terms of symptoms of chest tightness/pain and angina, with differing findings. Zhou et al. [56] found that patients with abnormal CT-FFR were significantly more likely to have symptoms of typical anginal chest pain and less likely to be asymptomatic. Zhang et al. [36] found that there was no significant difference in symptoms between the normal and abnormal CT-FFR groups, though this finding approached significance with a *p*-value of 0.05. These studies are both case–control studies; however, Zhang et al. [36] included MB patients with and without atherosclerosis, while Zhou et al. [56] included only patients without atherosclerosis.

Two studies reported on the diagnostic properties of CT-FFR for MB-related ischemia. The first study by Zhou et al. [38] used iFFR as the reference standard and reported a high PPV for CT-FFR in detecting ischemia in MB lesions with >70% proximal LAD stenosis on ICA, with low PPV for lesser degrees of stenosis. Despite this, CT-FFR maintained high sensitivity and NPV, regardless of the severity of stenosis, highlighting its use as an effective rule-out test for MB-related ischemia. In another study by Yu et al. [59], CT-FFR was compared against a reference standard of CT-MPI in patients with MB and no obstructive stenosis on CCTA. They found that CT-FFR had a high NPV but low PPV for MB-related ischemia, suggesting it as an effective rule-out test, with positive CT-FFR results requiring further evaluation by alternate modalities.

Moreover, while two studies showed a correlation between CT-FFR values and certain anatomical characteristics of MB [35,56] (suggesting that certain characteristics may in turn be more correlated to functionally significant MB lesions), Zhang et al. [36] found no significant difference in anatomical features in patients with normal and abnormal CT-FFR values. Again, these studies had different populations, with the former two including patients with no atherosclerosis and <50% coronary stenosis, respectively, while the latter included patients with and without atherosclerosis. In addition, a correlation between abnormal CT-FFR values and anatomical characteristics of MB alone does not imply an association between these characteristics and the functional significance of the MB, as CT-FFR has not been established as a validated tool for confirming MB-related ischemia.

Of note, the study by Jubran et al. [58] was the only study that included a comparison with dFFR, which, as previously described, has been considered the gold standard reference, specifically for the assessment of MB-related ischemia.

Beyond functional assessment, ML and radiomics models demonstrated predictive value for future plaque development, particularly in MB segments associated with altered hemodynamics. These findings suggest that AI may not only detect current disease but also anticipate downstream atherosclerotic risk.

Significant limitations across studies included retrospective study designs, single-center designs, methodological heterogeneity across studies, small sample sizes, varying reference standards or the lack of comparison to recognized reference standards for assessing ischemia or comparison to non-gold standard reference, the use of different CT-FFR techniques, the variable exclusion/inclusion criteria, and especially the variable inclusion of patients with atherosclerotic disease across studies, the lack of control groups in some studies, the lack of correlation with clinical data in most studies, and the lack of adjustment for confounding factors.

This review also has limitations. The search strategy was limited to English-language publications, and we did identify three Chinese-language publications during the search that were excluded. Moreover, the small number of eligible studies and their heterogeneity precluded quantitative synthesis and meta-analysis. Publication bias also cannot be excluded, and the findings may overrepresent positive results due to selective reporting.

Future research should focus on the prospective validation of AI-based tools in larger, multicenter cohorts using standardized imaging protocols and clinical endpoints. Integrating AI-derived functional parameters with clinical data, stress testing, or perfusion imaging may improve diagnostic accuracy and facilitate personalized management strategies. Furthermore, the incorporation of explainable AI may help bridge the interpretability gap, improving clinician trust and supporting regulatory adoption.

## 9. Conclusions

In conclusion, AI-enhanced cardiac CT, particularly through CT-FFR, shows promise in improving the assessment, characterization, and risk stratification of MB. Tools for the accurate detection of functionally relevant MB lesions are increasingly necessary in order to allow radiologists and clinicians to navigate the exponentially growing volume of highly accurate diagnostic CCTA studies and subsequent increase in detection of MB. While further validation is necessary, these technologies have the potential to transform current imaging paradigms by offering non-invasive, reproducible, and functionally meaningful assessments that can guide patient care more effectively.

## Figures and Tables

**Table 1:** Summary of AI techniques of interest for the assessment of MB.

	Purpose	Type	Requirements	Functional or Anatomical
AI-enhanced segmentation and reconstruction	Analysis of images to identify regions of interest, enhance image quality, and assess ischemia	Imaging + AI-based analysis	CT, MRI, or US images	Functional, anatomical
CFD	Simulate and analyze blood flow dynamics within vessels or the heart	Imaging + simulation	CT scan with contrast agent, computational modeling	Functional
CT-FFR	Assessment of the functional significance of stenosis by simulating blood flow	Imaging + simulation + ML-based analysis	CT scan with contrast agent, computational modeling	Functional

**Table 2:** Summary of studies included in the review.

Study	Journal	Country	Study Type	Study Aim	Population	Total Participants	Age	% Male	AI/ML Technique	Purpose of AI	Findings
Martens 2024 [55]	European Heart Journal—Case Reports	Belgium	Case report	To report on a case showing improvement in CT-FFR following treatment of MB	A patient with LAD MB and abnormal CT-FFR, which normalized after treatment with surgical unroofing of the MB	1	55	100	ML-based CT-FFR (HeartFlow)	Computation of CT-FFR	Normalization of CT-FFR from 0.76 pre-surgery to 0.92 post-surgery
Zhou 2019 [56]	European Radiology	China	Retrospective case–control	To evaluate the feasibility of CT-FFR derivation from CCTA in patients with MB, its relationship with MB anatomical features, and clinical relevance	Patients with LAD MB on CCTA with no atherosclerosis as compared with controls	161; 120 cases; 40 controls	Cases: 52.4 ± 11 Controls: 54.3 ± 11.5	Cases: 68 Controls: 54	ML-based CT-FFR (cFFR v3.0.0, Siemens Healthineers, Erlangen, Germany)	Computation of CT-FFR	MB is associated with abnormal CT-FFR values. MB length and systolic stenosis are the main contributors to abnormal CT-FFR values, with a combination of the two showing moderate predictive performance. Patients with abnormal FFR were less likely to be asymptomatic and more likely to have typical anginal chest pain
Zhou 2019 [57]	JACC: Cardiovascular Imaging	China	Retrospective cohort	To investigate the role of CT-FFR in predicting proximal plaque formation associated with MB in the LAD using ML approaches	Patients with MB in the LAD and no atherosclerosis on baseline CCTA who underwent follow-up CCTA with a minimum interval of 3 months	188	55 ± 6	68.6	ML-based CT-FFR (cFFR v3.0.0, Siemens Healthineers); ML-based prediction model using LASSO algorithms	Computation of CT-FFR; ML models for prediction of plaque formation	CT-FFR distal to LAD MB and ΔCT-FFR significantly differed between patients with CT MB LAD who developed plaque and those who did not. ML algorithms further identified CT-FFR and ΔCT-FFR as the strongest predictors of plaque formation proximal to MB LAD
Zhou 2019 [38]	Canadian Journal of Cardiology	China	Retrospective cohort	To study the diagnostic performance of ML-based CT-FFR to detect functional ischemia in MB with iFFR as the reference standard	Patients who underwent CCTA for the evaluation of suspected or known CAD and were found to have LAD MB who then underwent ICA within 60 days of CCTA	104	61.2 ± 9.1	72.1	ML-based CT-FFR (cFFR v3.2.0, Siemens Healthineers)	Automatic generation of centerline and luminal contours of coronary arteries; computation of CT-FFR	CT-FFR has high diagnostic performance for functional ischemia in vessels with MB and concomitant proximal atherosclerotic disease compared with iFFR, regardless of length and depth of MB, with a low PPV for lesions of <70% stenosis
Jubran 2020 [58]	Circulation: Cardiovascular Imaging	United States	Retrospective cohort	To compare CT-FFR, dobutamine-stress dFFR, iFFR, and IVUS in assessing the hemodynamic significance of MB	Patients with angina who had been found to have an MB in the LAD with ≤50% coronary artery stenosis by ICA and had undergone CCTA	CT-FFR: 49 dFFR: 43 iFFR: 28 IVUS: 46	47.5 ± 13.7	39	ML-based CT-FFR (cFFR v3.1.2, Siemens Healthineers)	Computation of CT-FFR	CT-FFR values measured in LAD MB were lower than arteries without MB. CT-FFR values did not correlate with dobutamine-stress dFFR, iFFR, or LAD systolic compression on IVUS. CT-FFR values were higher than dFFR and lower than iFFR. There was non-concordance between CT-FFR and dFFR, iFFR, or the degree of systolic compression measured by IVUS
Yu 2021 [59]	Korean Journal of Radiology	China	Cross-sectional	To investigate the diagnostic performance of CT-FFR for MB-related ischemia using dynamic CT-MPI as a reference standard	Symptomatic patients with LAD MB and no obstructive stenosis on CCTA who also underwent CT-MPI	75	62.7 ± 13.2	64	ML-based CT-FFR (cFFR; version 3.0, Siemens Healthineers)	Computation of CT-FFR	ΔCT-FFRsystolic shows high sensitivity and NPV and reliably excludes MB ischemia. All CT-FFR measurements had low PPV, and other methodologies are needed to confirm positive CT-FFR results
Zhang 2024 [37]	Clinical Radiology	China	Retrospective observational comparative study (within-subject)	To determine the effect of second-generation motion correction (MC2) on image quality and measurement reproducibility of CCTA images in patients with MB and mural coronary artery (MB-MCA) compared to standard (STD) images without motion correction and with first-generation motion correction (MC1)	Patients with known or suspected coronary artery disease who underwent CCTA and had MB-MCA in the LAD	66	62 ± 11	45	Deep learning image reconstruction algorithm (DLIR, GE Healthcare). First-generation motion correction algorithm (MC1, GE Healthcare). Second-generation motion correction algorithm (MC2, GE Healthcare)	Image reconstruction and motion correction	MC2 reduced motion artifacts and resulted in significant improvements of image quality, diagnostic confidence, and measurement reproducibility for both MB and MCA in systolic and diastolic phases
Zhang 2024 [36]	Clinical Physiology and Functional Imaging	China	Retrospective case–control	To quantitatively investigate the effect of MB in the LAD on CT-FFR	Patients with confirmed LAD MB on CCTA with or without atherosclerosis in the LAD as compared with controls	404; 300 cases; 104 controls	Cases: 54 ± 6 Controls: 54 ± 7	Cases: 56 Controls: 24	ML-based CT-FFR (Shukun, ct-FFR, V1.17)	Automatic extraction of coronary artery tree; automatic segmentation, reconstruction and diagnosis of coronary artery stenosis; computation of CT-FFR	No differences in CT-FFR values between systolic and diastolic phases. MB (with or without atherosclerosis) is associated with greater ΔCT-FFR and lower CT-FFR compared with controls without MB. CT-FFR is significantly lower in MB with atherosclerosis than in MB without atherosclerosis. In MB with atherosclerosis, LAD stenosis severity is an independent risk factor significantly affecting CT-FFR values, and abnormal CT-FFR (<0.80) is associated with more severe LAD stenosis. There was no significant difference detected in terms of clinical or anatomical features between the abnormal and normal CT-FFR groups
Chen 2024 [60]	European Heart Journal—Cardiovascular Imaging	China	Retrospective cohort and validation study	To develop and validate CCTA-based radiomics models in predicting proximal plaque development in LAD MB	Patients with MB and no atherosclerotic plaque proximal to the MB segment on baseline CCTA	295	55 ± 10	66	ML models	Predictive modelling and analysis	The proximal MB cross-sectional radiomics model (pMB CS) is able to predict proximal atherosclerotic plaque development associated with LAD MB in an external validation set (AUC 0.75; *p* < 0.001) and can be integrated with a clinical model to further improve performance (AUC 0.76; *p* < 0.001)
Sun 2024 [35]	Clinical Imaging	China	Cross-sectional	To compare the performance between CT-FFR and ΔCT-FFR in patients with deep LAD MB and explore predictors of discordance between the two measurements	Patients with deep LAD MB on CCTA and <50% stenosis of the LAD and/or left main stem	175	60 ± 7	71.4	Deep learning image reconstruction (TrueFidelity, GE Healthcare); ML-based CT-FFR (uAI Portal; United Imaging Intelligence)	Image reconstruction; automatic labeling of plaque and MB; computation of CT-FFR	A total of 30.9% of patients had discordance of CT-FFR and ΔCT-FFR, with 94.4% of patients leaning towards CT-FFR positivity with a negative ΔCT-FFR. Proximal atherosclerosis and distance from the MB to the aorta were independent risk factors for discordance. Anatomic features (length and depth) of the MB were correlated with ΔCT-FFR rather than CT-FFR, suggesting that ΔCT-FFR is a more specific tool for MB evaluation

## Data Availability

Full data extracted from the articles included in the literature review are made available by the authors upon request.

## References

[1] Rogers I.S., Tremmel J.A., Schnittger I. (2017). Myocardial bridges: Overview of diagnosis and management. Congenit. Heart Dis..

[2] Poláček P. (1961). Relation of myocardial bridges and loops on the coronary arteries to coronary occlusions. Am. Heart J..

[3] Ishii T., Asuwa N., Masuda S., Ishikawa Y. (1998). The effects of a myocardial bridge on coronary atherosclerosis and ischaemia. J. Pathol..

[4] Möhlenkamp S., Hort W., Ge J., Erbel R. (2002). Update on myocardial bridging. Circulation.

[5] Kawawa Y., Ishiwaka Y., Gomi T., Nagamoto M., Terada H., Ishii T., Kohda E. (2007). Detection of myocardial bridge and evaluation of its anatomical properties by coronary multislice spiral computed tomography. Eur. J. Radiol..

[6] Rubinshtein R., Gaspar T., Lewis B.S., Prasad A., Peled N., Halon D.A. (2013). Long-term prognosis and outcome in patients with a chest pain syndrome and myocardial bridging: A 64-slice coronary computed tomography angiography study. Eur. Heart J. Cardiovasc. Imaging.

[7] Feld H., Guadanino V., Hollander G., Greengart A., Lichstein E., Shani J. (1991). Exercise-induced ventricular tachycardia in association with a myocardial bridge. Chest.

[8] Nakanishi R., Rajani R., Ishikawa Y., Ishii T., Berman D.S. (2012). Myocardial bridging on coronary CTA: An innocent bystander or a culprit in myocardial infarction?. J. Cardiovasc. Comput. Tomogr..

[9] Ge J., Erbel R., Rupprecht H.J., Koch L., Kearney P., Görge G., Haude M., Meyer J. (1994). Comparison of intravascular ultrasound and angiography in the assessment of myocardial bridging. Circulation.

[10] Tonino P.A., De Bruyne B., Pijls N.H., Siebert U., Ikeno F., van’t V.M., Klauss V., Manoharan G., Engstrøm T., Oldroyd K.G. (2009). Fractional flow reserve versus angiography for guiding percutaneous coronary intervention. N. Engl. J. Med..

[11] Zimmermann F.M., Ferrara A., Johnson N.P., van Nunen L.X., Escaned J., Albertsson P., Erbel R., Legrand V., Gwon H.C., Remkes W.S. (2015). Deferral vs. performance of percutaneous coronary intervention of functionally non-significant coronary stenosis: 15-year follow-up of the DEFER trial. Eur. Heart J..

[12] Escaned J., Cortés J., Flores A., Goicolea J., Alfonso F., Hernández R., Fernández-Ortiz A., Sabaté M., Bañuelos C., Macaya C. (2003). Importance of diastolic fractional flow reserve and dobutamine challenge in physiologic assessment of myocardial bridging. J. Am. Coll. Cardiol..

[13] Angelini P., Uribe C., Raghuram A. (2025). Coronary Myocardial Bridge Updates: Anatomy, Pathophysiology, Clinical Manifestations, Diagnosis, and Treatment Options. Tex. Heart Inst. J..

[14] Ekeke C.N., Noble S., Mazzaferri E., Crestanello J.A. (2015). Myocardial bridging over the left anterior descending: Myotomy, bypass, or both?. J. Thorac. Cardiovasc. Surg..

[15] Tandar A., Whisenant B.K., Michaels A.D. (2008). Stent fracture following stenting of a myocardial bridge: Report of two cases. Catheter. Cardiovasc. Interv..

[16] Kunamneni P.B., Rajdev S., Krishnan P., Moreno P.R., Kim M.C., Sharma S.K., Kini A.S. (2008). Outcome of intracoronary stenting after failed maximal medical therapy in patients with symptomatic myocardial bridge. Catheter. Cardiovasc. Interv..

[17] Bamberg F., Marcus R.P., Becker A., Hildebrandt K., Bauner K., Schwarz F., Greif M., von Ziegler F., Bischoff B., Becker H.C. (2014). Dynamic myocardial CT perfusion imaging for evaluation of myocardial ischemia as determined by MR imaging. JACC Cardiovasc. Imaging.

[18] Ho K.T., Chua K.C., Klotz E., Panknin C. (2010). Stress and rest dynamic myocardial perfusion imaging by evaluation of complete time-attenuation curves with dual-source CT. JACC Cardiovasc. Imaging.

[19] Yang J., Dou G., He B., Jin Q., Chen Z., Jing J., Di Carli M.F., Chen Y., Blankstein R. (2020). Stress Myocardial Blood Flow Ratio by Dynamic CT Perfusion Identifies Hemodynamically Significant CAD. JACC Cardiovasc. Imaging.

[20] Li Y., Dai X., Lu Z., Shen C., Zhang J. (2021). Diagnostic performance of quantitative, semi-quantitative, and visual analysis of dynamic CT myocardial perfusion imaging: A validation study with invasive fractional flow reserve. Eur. Radiol..

[21] Schicchi N., Fogante M., Paolini E., Cela F., Pirani P.E., Perna G.P. (2023). Stress-rest dynamic-CT myocardial perfusion imaging in the management of myocardial bridging: A ‘one-stop shop’ exam. J. Cardiol. Cases.

[22] Williams M.C., Weir-Mccall J.R., Baldassarre L.A., De Cecco C.N., Choi A.D., Dey D., Dweck M.R., Isgum I., Kolossvary M., Leipsic J. (2024). Artificial intelligence and machine learning for cardiovascular computed tomography (CCT): A white paper of the society of cardiovascular computed tomography (SCCT). J. Cardiovasc. Comput. Tomogr..

[23] Slart R.H.J.A., Williams M.C., Juarez-Orozco L.E., Rischpler C., Dweck M.R., Glaudemans A.W.J.M., Gimelli A., Georgoulias P., Gheysens O. (2021). Position paper of the EACVI and EANM on artificial intelligence applications in multimodality cardiovascular imaging using SPECT/CT, PET/CT, and cardiac CT. Eur. J. Nucl. Med. Mol. Imaging.

[24] Sandeep B., Liu X., Huang X., Wang X., Mao L., Xiao Z. (2024). Feasibility of artificial intelligence its current status, clinical applications, and future direction in cardiovascular disease. Curr. Probl. Cardiol..

[25] Liao J., Huang L., Qu M., Chen B., Wang G. (2022). Artificial Intelligence in Coronary CT Angiography: Current Status and Future Prospects. Front. Cardiovasc. Med..

[26] Lopez-Jimenez F., Attia Z., Arruda-Olson A.M., Carter R., Chareonthaitawee P., Jouni H., Kapa S., Lerman A., Luong C., Medina-Inojosa J.R. (2020). Artificial Intelligence in Cardiology: Present and Future. Mayo Clin. Proc..

[27] Krittanawong C., Zhang H.J., Wang Z., Aydar M., Kitai T. (2017). Artificial Intelligence in Precision Cardiovascular Medicine. J. Am. Coll. Cardiol..

[28] Tolu-Akinnawo O.Z., Ezekwueme F., Omolayo O., Batheja S., Awoyemi T. (2025). Advancements in Artificial Intelligence in Noninvasive Cardiac Imaging: A Comprehensive Review. Clin. Cardiol..

[29] Kwan A.C., Salto G., Cheng S., Ouyang D. (2021). Artificial Intelligence in Computer Vision: Cardiac MRI and Multimodality Imaging Segmentation. Curr. Cardiovasc. Risk Rep..

[30] Wu Y., Tang Z., Li B., Firmin D., Yang G. (2021). Recent Advances in Fibrosis and Scar Segmentation From Cardiac MRI: A State-of-the-Art Review and Future Perspectives. Front. Physiol..

[31] Alnasser T.N., Abdulaal L., Maiter A., Sharkey M., Dwivedi K., Salehi M., Garg P., Swift A.J., Alabed S. (2024). Advancements in cardiac structures segmentation: A comprehensive systematic review of deep learning in CT imaging. Front. Cardiovasc. Med..

[32] Liang J., Sun Y., Ye Z., Sun Y., Xu L., Zhou Z., Thomsen B., Li J., Sun Z., Fan Z. (2019). Second-generation motion correction algorithm improves diagnostic accuracy of single-beat coronary CT angiography in patients with increased heart rate. Eur. Radiol..

[33] Sun J., Okerlund D., Cao Y., Li H., Zhu Y., Li J., Peng Y. (2020). Further improving image quality of cardiovascular computed tomography angiography for children with high heart rates using second-generation motion correction algorithm. J. Comput. Assist. Tomogr..

[34] Joshi M., Melo D.P., Ouyang D., Slomka P.J., Williams M.C., Dey D. (2023). Current and Future Applications of Artificial Intelligence in Cardiac CT. Curr. Cardiol. Rep..

[35] Sun Q., Zhang J., Wang W., Qi Y., Lyu J., Zhang X., Li T., Lou X. (2024). Predictors of discordance between CT-derived fractional flow reserve (CT-FFR) and △CT-FFR in deep coronary myocardial bridging. Clin. Imaging.

[36] Zhang D., Tian X., Li M.Y., Zheng W.S., Yu Y., Zhang H.W., Pan T., Gao B.L., Li C.Y. (2024). Quantitative computed tomography angiography evaluation of the coronary fractional flow reserve in patients with left anterior descending artery myocardial bridging. Clin. Physiol. Funct. Imaging.

[37] Zhang Z., Liu Z., Hong N., Chen L. (2024). Effect of a second-generation motion correction algorithm on image quality and measurement reproducibility of coronary CT angiography in patients with a myocardial bridge and mural coronary artery. Clin. Radiol..

[38] Zhou F., Zhou F., Wang Y.N., Schoepf U.J., Tesche C., Tang C.X., Zhou C.S., Xu L., Hou Y., Zheng M.W. (2019). Diagnostic Performance of Machine Learning Based CT-FFR in Detecting Ischemia in Myocardial Bridging and Concomitant Proximal Atherosclerotic Disease. Can. J. Cardiol..

[39] Gijsen F., Katagiri Y., Barlis P., Bourantas C., Collet C., Coskun U., Daemen J., Dijkstra J., Edelman E., Evans P. (2019). Expert recommendations on the assessment of wall shear stress in human coronary arteries: Existing methodologies, technical considerations, and clinical applications. Eur. Heart J..

[40] Stone P.H., Maehara A., Coskun A.U., Maynard C.C., Zaromytidou M., Siasos G., Andreou I., Fotiadis D., Stefanou K., Papafaklis M. (2018). Role of Low Endothelial Shear Stress and Plaque Characteristics in the Prediction of Nonculprit Major Adverse Cardiac Events. JACC Cardiovasc. Imaging.

[41] Kumar A., Thompson E.W., Lefieux A., Molony D.S., Davis E.L., Chand N., Fournier S., Lee H.S., Suh J., Sato K. (2018). High. Coronary Shear. Stress. in Patients With Coronary Artery Disease Predicts Myocardial Infarction. J. Am. Coll. Cardiol..

[42] Candreva A., De Nisco G., Rizzini M.L., D’Ascenzo F., De Ferrari G.M., Gallo D., Morbiducci U., Chiastra C. (2022). Current and Future Applications of Computational Fluid Dynamics in Coronary Artery Disease. Rev. Cardiovasc. Med..

[43] Wojtas K., Kozłowski M., Orciuch W., Makowski Ł. (2021). Computational Fluid Dynamics Simulations of Mitral Paravalvular Leaks in Human Heart. Materials.

[44] Fezzi S., Ding D., Scarsini R., Huang J., Del Sole P.A., Zhao Q., Pesarini G., Simpkin A., Wijns W., Ribichini F. (2023). Integrated Assessment of Computational Coronary Physiology From a Single Angiographic View in Patients Undergoing TAVI. Circ. Cardiovasc. Interv..

[45] Javadzadegan A., Moshfegh A., Fulker D., Barber T., Qian Y., Kritharides L., Yong A.S.C. (2018). Development of a Computational Fluid Dynamics Model for Myocardial Bridging. J. Biomech. Eng..

[46] Sharma P., Itu L., Zheng X., Kamen A., Bernhardt D., Suciu C., Comaniciu D. A framework for personalization of coronary flow computations during rest and hyperemia. Proceedings of the 34th Annual International Conference of the IEEE Engineering in Medicine and Biology Society.

[47] Gulati M., Levy P.D., Mukherjee D., Amsterdam E., Bhatt D.L., Birtcher K.K., Blankstein R., Boyd J., Bullock-Palmer R.P., Conejo T. (2021). 2021 AHA/ACC/ASE/CHEST/SAEM/SCCT/ SCMR Guideline for the Evaluation and Diagnosis of Chest Pain: A Report of the American College of Cardiology/American Heart Association Joint Committee on Clinical Practice Guidelines. Circulation.

[48] Bittl J.A., Bangalore S., DiMaio J.M., Grant M.C., Lawton J.S., Tamis Holland J.E. (2022). Putting the 2021 ACC/AHA/SCAI Guideline for Coronary Artery Revascularization Into Practice. JACC Case Rep..

[49] Neumann F.-J., Sousa-Uva M. (2019). ‘Ten commandments’ for the 2018 ESC/EACTS Guidelines on Myocardial Revascularization. Eur. Heart J..

[50] Yang S., Chung J., Lesina K., Doh J.H., Jegere S., Erglis A., Leipsic J.A., Fearon W.F., Narula J., Koo B.K. (2024). Long-term prognostic implications of CT angiography-derived fractional flow reserve: Results from the DISCOVER-FLOW study. J. Cardiovasc. Comput. Tomogr..

[51] Nørgaard B.L., Leipsic J., Gaur S., Seneviratne S., Ko B.S., Ito H., Jensen J.M., Mauri L., De Bruyne B., Bezerra H. (2014). Diagnostic performance of noninvasive fractional flow reserve derived from coronary computed tomography angiography in suspected coronary artery disease: The NXT trial (Analysis of Coronary Blood Flow Using CT Angiography: Next Steps). J. Am. Coll. Cardiol..

[52] Rajiah P., Cummings K.W., Williamson E., Young P.M. (2022). CT Fractional Flow Reserve: A Practical Guide to Application, Interpretation, and Problem Solving. RadioGraphics.

[53] Tesche C., De Cecco C.N., Albrecht M.H., Duguay T.M., Bayer R.R., Litwin S.E., Steinberg D.H., Schoepf U.J. (2017). Coronary CT Angiography–derived Fractional Flow. Reserve. Radiol..

[54] Seetharam K., Brito D., Farjo P.D., Sengupta P.P. (2020). The Role of Artificial Intelligence in Cardiovascular Imaging: State of the Art Review. Front. Cardiovasc. Med..

[55] Martens B., Michiels V., Argacha J.F., Cosyns B. (2024). Normalization of FFR_CT_ after surgical unroofing of a myocardial bridge: A case report. Eur. Heart J. Case Rep..

[56] Zhou F., Tang C.X., Schoepf U.J., Tesche C., Bauer M.J., Jacobs B.E., Zhou C.S., Yan J., Lu M.J., Lu G.M. (2019). Fractional flow reserve derived from CCTA may have a prognostic role in myocardial bridging. Eur. Radiol..

[57] Zhou F., Tang C.X., Schoepf U.J., Tesche C., Rollins J.D., Liu H., Zhou C.S., Yan J., Lu M.J., Lu G.M. (2019). Machine Learning Using. CT-FFR Predicts Proximal Atherosclerotic Plaque Formation Associated With LAD Myocardial Bridging. JACC Cardiovasc. Imaging.

[58] Jubran A., Schnittger I., Tremmel J., Pargaonkar V., Rogers I., Becker H.C., Yang S., Mastrodicasa D., Willemink M., Fleischmann D. (2020). Computed Tomographic Angiography-Based Fractional Flow. Reserve Compared With Catheter-Based Dobutamine-Stress. Diastolic Fractional Flow. Reserve in Symptomatic Patients With a Myocardial Bridge and No Obstructive Coronary Artery Disease. Circ. Cardiovasc. Imaging.

[59] Yu Y., Yu L., Dai X., Zhang J. (2021). CT Fractional Flow Reserve for the Diagnosis of Myocardial Bridging-Related Ischemia: A Study Using Dynamic CT Myocardial Perfusion Imaging as a Reference Standard. Korean J. Radiol..

[60] Chen Y.C., Zheng J., Zhou F., Tao X.W., Chen Q., Feng Y., Su Y.Y., Zhang Y., Liu T., Zhou C.S. (2024). Coronary CTA-based vascular radiomics predicts atherosclerosis development proximal to LAD myocardial bridging. Eur. Heart J. Cardiovasc. Imaging.

